# Am I (Deep) Blue? Music-Making AI and Emotional Awareness

**DOI:** 10.3389/fnbot.2022.897110

**Published:** 2022-06-21

**Authors:** Nicholas Novelli, Shannon Proksch

**Affiliations:** ^1^Independent Researcher, Winnipeg, MB, Canada; ^2^Cognitive and Information Sciences, University of California, Merced, Merced, CA, United States

**Keywords:** music cognition, artificial intelligence, emotion, interoception, creativity, aesthetics

## Abstract

Artificial Intelligence has shown paradigmatic success in defeating world champions in strategy games. However, the same programming tactics are not a reasonable approach to creative and ostensibly emotional artistic endeavors such as music composition. Here we review key examples of current creative music generating AIs, noting both their progress and limitations. We propose that these limitations are rooted in current AIs lack of thoroughly embodied, interoceptive processes associated with the emotional component of music perception and production. We examine some current music-generating machines that appear to be minimally addressing this issue by appealing to something akin to interoceptive processes. To conclude, we argue that a successful music-making AI requires both the generative capacities at which current AIs are constantly progressing, and thoroughly embodied, interoceptive processes which more closely resemble the processes underlying human emotions.

## 1. Introduction

In the race to build increasingly autonomous–perhaps even conscious–machines, focus on machine learning and machine intelligence is on the rise. Paradigmatic AI successes in games such as chess and Go have relied heavily on computational processes that occur primarily “in the head” of game-playing agents. 4E (embodied, embedded, enactive, extended) approaches to cognition are increasingly demonstrating the importance of cognitive processes which extend beyond such rule-based symbol manipulation, and into the bodies and external environments of cognitive agents. The next great frontier for autonomous intelligent systems is human creativity and art. Specifically, an art form that encapsulates the tenets of 4E cognition and places an emphasis on the agent's interaction with their social environment, as well as their external and internal milieu: music. Numerous music-making AIs have been created in attempts to simulate, understand, or replicate the process of human creativity in musical composition using artificial neural networks, such as Google's Magenta, Cambridge University's BachBot, or Sony CSL's Flow Composer.

There are practical reasons that computers have difficulty performing creative tasks as successfully as strategic tasks, due to both mathematical complexity and a deep connection with emotional processing in human music-making. These emotional processes have roots in bodily, physiological, and autonomic states in the performer and the listener. We draw on theories that emphasize thoroughly embodied, interoceptive processes rooted in the prediction and regulation of internal physiological processes as part of the mechanism of human emotion (Seth and Friston, 2016), and extend these theories to musical perception and production (Proksch, 2018). If these bodily processes are crucial to creative musical success, then AIs will need such mechanisms (or analogues to them) in order to create authentic music. We argue that generative music AIs must experience, or robustly simulate, something akin to the interoceptive processes that underlie emotional states.

## 2. Artificial Intelligence: Defeating Champions, Approximating Music

### 2.1. AI Successes: Defeating Champions

#### 2.1.1. DeepBlue and AlphaGo

If AI Success is measured in terms of the ability to equal (or outperform) expert human counterparts, then IBM's Deep Blue and Google's AlphaGo are paradigmatic successes. DeepBlue beat Gary Kasparov in 1997 by mapping every possible combination of moves it could make, up to the next six moves, according to a set of pre-programmed rules and evaluations established with the help of expert chess players (Campbell, 1998). Nearly 20 years later, AlphaGo beat Lee Sedol, the world champion of the strategy game “Go.”

DeepBlue and AlphaGo both used a directed graph, called a game tree, which represents possible moves and positions for multiple sequences of game play. A complete game-tree for chess would contain 10^120^ moves (already more than the number of atoms in the universe, 10^80^), and a game-tree for Go would massively exceed this number. Before AlphaGo, most Go AIs achieved this computational feat by using a technique called Monte Carlo Tree Search. Instead of looking through pre-programmed mappings of every possible combination of moves from a current state, the AI stores only the rules, and runs multiple simulations extending from the current state until any winning state, re-running this simulation for every move. AlphaGo used machine learning combined with policy and value (neural) networks to determine the move with the highest likelihood of a win, in combination with simulative tree-search methods (Silver et al., 2016). Using a variety of methods, strategy game AIs have achieved expert-level success. Given AI successes at strategy games, one might wonder if these techniques extend to creative (albeit rule-based) endeavors such as music-making. Could this strategy work for music? Music composition has far more possibilities at each decision-point than chess or Go, and does not have rules in the same sense of options being forbidden and thus eliminated (many of our most beloved songs break a supposed “rule” of composition; Cochrane, 2000), nor success or failure states that end the exercise early.

It is impractical to even store a “tree” of all possible musical sequences of any reasonable length, let alone to encode any information about their aesthetic viability. A song might range across two or more octaves, each with twelve notes and 60 possible chords for each instrument part, plus differences in rhythm, tempo, orchestration, to say nothing of going beyond the traditional Western paradigm, e.g., including quarter-tones. We need a shortcut. Current music-making AIs have attempted to achieve this shortcut in ways similar to AlphaGo, using recurrent neural networks with value programmed as the probabilistic likelihood of the next note, given the structural information of the previous notes in terms of rhythm, pitch, etc. These AIs have produced promising results (as we shall see) but the evidence is growing that there is a limit to what can be accomplished with probabilistic structural data of the musical input alone.

### 2.2. AI Works in Progress: Approximating Music

Music-generating AIs work primarily by learning patterns in the structural information-pitch, rhythm, harmony, etc. of a set of musical training data. After training, the AI is provided with a set of rules and some form of starting cue from which it generates a piece of music. There are many different methods one can use to create a music generating AI, reviewed in detail in Carnovalini and Rodà (2020). Here, we will focus on three examples of generative AIs which learn from musical training input to probabilistically generate musical compositions.

#### 2.2.1. Magenta

Magenta is a far-reaching Google project that seeks to determine whether machine learning can be used to create “compelling art and music”^1^. Magenta's early music compositions used Recurrent Neural Networks (RNNs), which work by learning a probability distribution of possible inputs given previous data, in order to predict the next input. After a series of training data, the RNN can now generate its own output using the same probabilistic rules. Magenta is trained on thousands of monophonic (single note at a time) melodies, from which it learns the rules and style of those melodies, and develops probabilistic models which it uses to generate new monophonic melodies on its own.

The result, in Magenta's first ever composition in June 2016, was a fairly impressive, albeit simplistic composition, reminiscent of a standard theme and variations^2^. Just under one and one-half minutes long, it begins with a simple but clear motif set to a repeating eighth-note and quarter-note rhythm, which repeats first verbatim, then again with a bit of clumsy ornamentation, before entering a “creative” development section introducing some awkward new rhythms. The piece returns to the original motif, before ultimately and abruptly stopping mid-phrase with no clear conclusion.

This abrupt ending occurs because, unlike in a human composition, many music-making AIs thus far have no concept of a musical “narrative arc” with conclusive resolutions. Instead, they will tend to “wander around” their music-making process unless a human programs it to stop at a certain point. Much of Magenta's work thus far has dealt with monophonic compositions, generating a single note at a time^3^. This contrasts with the next examples that use multiple streams of notes.

#### 2.2.2. BachBot

At first listen, BachBot's^4^ compositions are much more impressive than the debut single-note melody from the Magenta Project. BachBot also uses Long-Short Term Memory (LSTM) RNNs, and is trained specifically on Bach chorales. Unlike Magenta, its training input is homophonic, or chordal, with a series of simultaneous pitches (chords) organized as melody plus harmony, all composed by J.S. Bach. Like Magenta, BachBot used its probabilistic models of which chords should come next to generate new pieces (here in the style of Bach chorales) on its own. The music created by this AI is much more sophisticated than the melodic-play generated by Magenta, and to the untrained ear is virtually indistinguishable from that composed by Bach himself^5^.

Unlike Magenta's rough approximation of a theme and variations, these chorales follow a cadential structure with well-organized phrases^6^. Instead of awkwardly-placed ornaments, BachBot's chorales contain purposeful passing tones within a stable rhythmic structure. However, while impressive, BachBot suffers some of the same problems as Magenta. Unless Bachbot is given at least one line of a chorale (or a melody) to harmonize over, it will suffer the same “wandering” fate as Magenta's compositions. It maintains its semblance of structure because a human provides it with a prescribed line of notes, which then constrains its output and leads BachBot through a structured journey of composition.

#### 2.2.3. Flow Composer (Paris, Sony Computer Science Laboratories, ERC Funded Project

The even more impressive Flow Composer^7^ was created to produce pop songs. Rather than LSTM's, Flow Composer uses Markov constraints. This solves the “wandering” problem faced by both Magenta and Bachbot by generating finite-length sequences, and similar to Bachbot it generates these sequences in accordance with a given composer-style, or genre of music. Flow Composer takes input for model generation in the form of lead sheets (basic chord structure plus a melody line), and once again uses its probabilistic memory to generate a new lead sheet for a new song all on its own.

Flow Composer created the first ever full-length pop song composed by an AI, the Beatles-“inspired” track “Daddy's Car”^8^. Daddy's Car has lyrics, with multiple voices, guitar, drums—a full orchestration. However, as impressive as this is, and despite the problems apparently solved from Magenta and Bachbot dealing with wandering and improper ornamentation or rhythm, the only thing that Flow Composer generates is a lead sheet. The rest of the music composing process, including writing the harmonies themselves, instrumentation, and writing the lyrics, are performed by human collaborators^9^.

### 2.3. What's Missing?

There is a common thread amongst these music-making AIs, and that is the importance of the human in the process. This is partially rooted in the fact that each example is not truly generating musical content, but is reliant to some degree on human intervention. In fact, Magenta, with the most basic and least impressive of the compositions highlighted here, composes music with the least help from human musical decisions. If each composition was subjected to a sort of musical “Turing test,” Magenta's might be the least likely to pass because it rests in an “uncanny valley” between quality music and childlike—or just plain strange—artificial creativity. However, the other compositions might pass solely due to the human intervention necessary to yield the final musical product. It might be countered that this is simply a difference of degree, because human composers are still better at music than our AIs. But at what, exactly, are humans better?

Although music students and young musicians are taught and trained in the rules and norms of their musical culture, there is a common pre-theoretical or folk-psychological notion that what is important in composing music is the expression or elicitation of emotion. Good music does not just blindly follow rules, it has feeling, emotion. Historical and current work in music cognition indicates that part of what enables humans to both process and create music in the way that we do involves inherently emotional processes (Huron, 2008; Juslin and Västfjäll, 2008; Trost et al., 2012; Koelsch et al., 2015). Current trends in the philosophy of cognitive science indicate these emotional processes are rooted in the prediction and regulation of internal physiological processes, or interoceptive states. Conceiving of the experience of emotional states as crucially involving interoceptive processing has important implications for music-making AIs.

## 3. Emotion and Interoception

There are competing accounts of what makes an “emotion,” however all accounts consider the importance or interoceptive, physiological states of the body. If an emotional experience arises from gathering evidence from the state of our body, plus a subsequent-or simultaneous-cognitive appraisal (James, 1884; Lange, 1885; Schachter and Singer, 1962), then the brute-force rule-following and simulation-based success of strategy game AIs could be extrapolated to emotional and creative processes like music making. There are some reliable cross cultural mappings of particular musical sounds to particular (potentially emotional) functions—such as the downward melodic passages and slow rhythms of soothing lullabies (Mehr et al., 2019). Indeed, musicians make use of standard motifs within their musical traditions that are associated with or meant to evoke certain emotions in an audience. An AI could, in theory, form a reliable mapping between statistical regularities of music and emotion across cultures, even without a physical body to instantiate those interoceptive processes itself.

However, making music that elicits or evokes an appropriate emotion is not as simple as choosing from a library of sound sequences coded for emotional content. Emotional experience relies on expectations about the way that interoceptive states of the body will unfold with respect to the external and social context of that experience (Critchley, 2005; Seth, 2013; Seth and Critchley, 2013). In this vein, making music with emotion relies on expectations about the way that interoceptive states of the body will respond to music-listening and music-making. In fact, experience with the bodily movement involved in making and moving music leads to enhanced interoceptive awareness for both musicians and dancers alike (Schirmer-Mokwa et al., 2015; Christensen et al., 2018). Information from relevant interoceptive states (whether first-hand, i.e., having a body capable of them, or second-hand, i.e., interacting with an individual that does), can enhance artificial music generation systems' ability to create compelling music.

The experience of emotion in music listening and music production is rooted in expectancy of not just the structural information of music (for which our AIs are very capable), but also more thoroughly embodied expectancy of the internal, physiological state which is either cued or expressed in music listening or creating, respectively (Proksch, 2018). Music listening is a common tool by which individuals monitor and regulate their emotional states and supporting neurochemistry (Chanda and Levitin, 2013). Consider the practice of listening to calming music as you fall asleep—calming because it cues the brain to minimize levels of cortisol and adrenaline in your body (McKinney et al., 1997; Khalfa et al., 2003; Thoma et al., 2013). Or the opposite, listening to upbeat energetic music on your morning run, inciting increased general arousal, and enjoyment of physical exertion (jymming', c.f. Fritz et al., 2013a,b), while the exercise itself may even boost increased enjoyment of the music (Hove et al., 2021). These same processes are leveraged by a composer, or an improviser, who is creating music in response to or in order to modulate their audience's emotional, and by extension physiological, states.

The exteroceptive information of the music, the structural organization of pitch and rhythm, is mutually contextualized by the thoroughly embodied, interoceptive information which is used to generate the music itself, and the integration of these two forms of information in the creative process of music composition is what leads to the pre-theoretical intuition that may be deemed the “heart and soul” of a musical work. Music-making AIs, and the music they compose, are thought to lack this emotional quality. Since computer programs lack the proper embodied, interoceptive states and homeostatic physiological drive, by which emotions are proposed to be constituted, then this pre-theoretical intuition is plainly justified. It seems that music-making AIs cannot compose authentic music because they lack the interoceptive, emotional processes necessary to do so.

Thus far, we have observed that the most musically impressive programs have a higher degree of human intervention to achieve a satisfying musical structure. We have justified criticisms of AI's musical output by demonstrating that the pre-theoretical notion that computer composed music lacks the “heart and soul” of authentic, human composed music can be rooted in basic interoceptive processes of physiological homeostasis. By incorporating this more thoroughly embodied process, AIs may be able to avoid their worst tendencies and ground their musical output in terms of their own interoceptive states, and come closer to attaining states resembling something like human emotions. In this next section, we will present two more music machines which may create closer approximations to authentic musical works.

### 3.1. Minimally Interoceptive Artificial Music Generation

#### 3.1.1. Magenta: AI Duet

We return to the Magenta project to visit an interactive, improvising music machine. AI Duet runs on similar models as we've discussed earlier, using RNNs and LSTMs to learn the rules and styles of its input, and then using those rules to generate its own musical output. However, this time, AI Duet takes input from a human, improvising musician—in real time—and together they improvise their own joint musical performance. Simply put, when you play a series of notes, the computer will respond to those notes, sometimes mimicking, mirroring, or expanding on the input you've given it. This is much more impressive than Magenta's initial attempts at music composition, in that it incorporates real time social interaction. The music generated by these social performances range from an awkward situation between two mediocre or completely inexperienced improvisers, to fairly convincing collaborative experiments. These experiments, whether or not they are aesthetically pretty or pleasing musically, have some semblance of feeling. This is because the musical event as a whole, in this case, is partially rooted in interoceptive processes—albeit only the embodied interoceptive processes of the human collaborator. The computer program itself is still only processing the exteroceptive content of the musical structure. If two of these music AIs interact together, the music produced quickly becomes nonsensical in the same way that the conversation between two chatbots quickly deteriorates.

#### 3.1.2. Cybraphon

Designed in 2009 by the FOUND artist collective together with Simon Kirby from the University of Edinburgh, Cybraphon is a “moody, autonomous robot band in a box” and is housed at the National Museum of Scotland (National Museums Scotland, 2009). The instrument is quite literally a wardrobe, filled with musical objects, lights, an “emotion meter,” and a computer which controls when each of these objects will sound, light up, or move (Taubman, 2014). Unlike the previous AIs discussed, Cybraphon is not entirely generative from the ground up. Rather, it performs by choosing from a repertoire of precomposed bits of music, and selects the music that corresponds to its current “emotional” state. This “emotional state” is not a product of solving the problem of giving a robot a homeostatic body, but rather from being thoroughly entrenched in social media. Cybraphon is a bit of a diva—it Googles itself every fifteen seconds and observes its current popularity over news sites, twitter, and facebook. The more online activity, and the more positive the online activity, Cybraphon will “cheer up” and might play one of its happy tunes. If no online interaction is happening, it will sit in a state of perpetual indifference, refusing to make music at all^10^. Cybraphon has something like extended interoceptive processing, loosely embodied by activity of the online community. Although similar to AI Duet's reliance on human interoceptive processes in the creation of a musical event, Cybraphon does not rely on any one person or group of persons' interoceptive processes, but rather translates social media activity into loosely embodied “emotional” states based on the online activity's deviation from normal levels. This nearly resembles the sort of embodied process in which interoceptive emotions are proposed to be rooted. However, the instrument lacks a predictive component that might enable it to probabilistically seek a homeostatic set point for these extended interoceptive states.

### 3.2. What's Missing

Crucially, the prediction and regulation of interoceptive states relies to some extent on bodily action. In fact, the very language of *seeking* a homeostatic set point to bring about an interoceptive and emotional state implies that an embodied music-making AI must be able to take action in the world to affect its own internal states. Magenta AI:Duet, while minimally interactive, does not have a body to take action in the world or interoceptive processes to respond to the rhythmic and melodic content that is co-generated by the AI and human performer. It relies on the actions of the human duet partner. Cybraphon does take some action in the world through the small repertoire of mechanical actions it can make in response to its extended interoceptive state, which itself depends on engagement of others in the world. However, Cybraphon's own actions have no effect on its interoceptive states, and it cannot interact with other individuals during its music making. The ability to act on interoceptive processes, and interact with other individuals, may be one more ingredient missing for successful music generation by music-making machines.

## 4. Action and Interaction

### 4.1. Movement in Music Generating Robots

Human music-making is, itself, “inseparable” from movement (Keller and Rieger, 2009). Even passive music listening is strongly rooted in motor processes in the brain (Grahn and Brett, 2007; Gordon et al., 2018). Anticipation of melodic, harmonic, and rhythmic content of a musical work engages canonical emotion, reward, and motor networks in the brain (Salimpoor et al., 2015; Vuust et al., 2022). Rhythmic components of music are acutely associated with predictive and motor processes (Koelsch et al., 2019; Proksch et al., 2020). In particular, there is a human urge to move to a musical beat that may be strongly related to the balance of sensory prediction and prediction error elicited by rhythmic syncopation found in musical groove, and higher levels of musical groove are rated as more pleasurable (Janata et al., 2012; Witek et al., 2014). Joint movement to musical rhythm can result in the co-activation of motor networks related to the perception of self and other (Overy and Molnar-Szakacs, 2009; Friston and Frith, 2015), engaging the endogenous opioid system and mirroring mechanisms which support social bonding (Tarr et al., 2014). There has been increasing recognition of music as an inherently enactive and interactive process, mutually co-constituted in the actions of musicians predicting both musical (exteroceptive) and bodily sensations (interoceptive and proprioceptive states) (Cross, 2014; Dell'Anna et al., 2021). Joint musical interaction is further aided by visual (exteroceptive) information regarding the movement, intention, and interest of each musician in addition to internal representations of movements of the other interacting musicians (Novembre et al., 2012, 2014). We next provide an example of an embodied and interactive music-making AI—an improvising, marimba-playing robot.

### 4.2. Shimon

Created by the Robotic Musicianship Group at Georgeia Tech Center for Musical Technology, Shimon is trained on an extensive repertoire of classical, jazz, and popular music. Similar to Magenta AI:Duet, Shimon is a music-making AI that engages in musical improvisation alongside human performers. However, Shimon is physically embodied in a marimba playing robot with four arms that can play melodic, rhythmic, and multiphonic music (Weinberg et al., 2009). Further, this robot features an expressive “face” that can move along with a musical beat and facilitate interaction with ensemble musicians^11^. Similar embodied robotic music machines have been designed to play traditional instruments, such as piano, violin, and flute as well as new forms of musical instruments afforded by the different physical configurations a musical robot can take compared to humans (Bretan and Weinberg, 2016). These robots create “a sense of embodiment” that afford “richer musical interactions” between human musicians and robotic music-making AIs (Bretan and Weinberg, 2016). For instance, Shimon nods along to the musical beat—mimicking the human propensity to move to a beat—and direct its attention by turning its head toward the musician playing the most interesting (i.e., salient) musical line when interacting in a musical ensemble (Weinberg et al., 2009). The actions taken by the robots may not refer to any interoceptive or emotional state internal to the robot, but may be robustly simulating such states through their actions so as to facilitate self-other merging and social emotions among their human co-performers.

## 5. Discussion and Conclusion

### 5.1. Additional Considerations: Musico-Historical and Social Context

There are other aesthetic properties that an even more embodied, embedded, environmentally interactive form of AI might succeed at producing. Music can be judged not only by how it makes us feel and how pleasant it sounds, but on the basis of properties like innovativeness, subversiveness, homage to other works, etc. For an AI to master these properties, it would have to have an awareness of musico-historical context, beyond mere probability distributions over the notes, rhythms, and features of a particular song or musical style. Jerrold Levinson enumerates musico-historical context as some personal components (a composer's own style, repertoire, oeuvre, and influences), and some general components (the history of musical development, prevalent musical styles, and influences at time of composition, and activities of contemporary composers) (Levinson, 1980). A musical AI might need to be socially embedded within a musico-historical context to have mastery of these complex—and even some more simple—aesthetic properties. A composer does not rely on her own feelings alone, and imagine if Cybraphon could not only monitor social media reactions, but also processed the nature of positive and negative critiques and tracked exactly which aspects of its compositions some listeners find annoying or sublime. But a more robust system wherein an A.I. composer is educated by an artistic community could develop the ability to create beyond its teachers or its training data, and play off the works of others in a way that adds aesthetic depth^12^.

All this might require engaging and participating in a musical community rather than simply processing data.

#### 5.1.1. Spawn

One such system has been created by Holly Herndon. The singing AI called “Spawn” was trained on her own voice, the voices of her musical collaborators (Friedlander, 2019), and even the voices of her audience (Herndon, 2019). Herndon says that as opposed to AIs such as Bachbot that make music in one particular style, her goal was to create an AI that can “understand the logic of a sound sample” and thus be more adaptable (Friedlander, 2019). A strong emphasis of the project is the “raising” of an AI by a “community.” Herndon is careful to be transparent about the limitations of the technology and estimates the contribution of the AI in each musical composition at about twenty percent.

### 5.2. The Future of Music Machines

To conclude, while music-making AI is thriving on the progress we have made in generative music machines, something is yet missing. Music-making AIs are unable to reach success by relying on brute-force rule memorization and future state simulation in the same manner as competitive strategy game AIs. Successful compositions by music-making AIs thus far, while appearing autonomously generative, have required a significant amount of human intervention. Even with this intervention, these compositions seem to be lacking feeling, emotion, and a focused narrative structure. We demonstrated that human music production and perception is not merely “in the head,” but rather involves influence from homeostatic, interoceptive processes in which human emotion processing is grounded. This interoceptive processing is importantly lacking in computer programs creating musical compositions. Two music-machines, AI Duet and Cybraphon appear to be minimally incorporating a form of interoceptive processing, however the former is reliant upon input from a human collaborator, and the latter is not generative. Current music-making robots, such as Shimon, may be more adept at mimicking actions which, when made by a human, are rooted in emotional and interoceptive processes—enabling rich musical interactions as a member of a musical ensemble. Spawn is an example of a budding musical AI which is raised by and embedded in a community, learning, and evolving through interactions with humans rather than from pre-composed datasets of music. A successful music-making AI will need to build on current generative successes, and incorporate more thoroughly embodied interoceptive processing of a sort that would ground the machine's musical output to its own internal, perhaps even conscious, states. Essentially, they must be able to ask themselves, “Am I Blue?.”

## Data Availability Statement

The original contributions presented in the study are included in the article/supplementary material, further inquiries can be directed to the corresponding author/s.

## Author Contributions

NN contributed expertise on artificial intelligence, cyborg technology, aesthetics, and philosophy of music. SP contributed expertise on music cognition, 4e processing, interoception, and machine learning and neural networks. Both authors contributed to writing and revision of all aspects of the manuscript, and approved the submitted version.

## Conflict of Interest

The authors declare that the research was conducted in the absence of any commercial or financial relationships that could be construed as a potential conflict of interest.

## Publisher's Note

All claims expressed in this article are solely those of the authors and do not necessarily represent those of their affiliated organizations, or those of the publisher, the editors and the reviewers. Any product that may be evaluated in this article, or claim that may be made by its manufacturer, is not guaranteed or endorsed by the publisher.

## Footnotes

^1^Magenta is an open source project from the Google Brain team.Source code and updates can be found at: https://github.com/tensorflow/magenta.

^2^To listen to Magenta's first composition: https://www.youtube.com/watch?v=lht-emTioLw.

^3^There has been improvement in establishing a narrative structure by implementing hierarchical models such as MusicVAE. MusicVAE implements a variational autoencoder consisting of a bidirectional LSTM network and a novel hierarchical RNN decoder. This allows for generation of music sequences with a more coherent long-term structure. Listen to MusicVAE at: https://magenta.tensorflow.org/music-vae.

^4^BachBot is also open source, run by researchers at the University of Cambridge and Microsoft Research Center. You can find source code and updates at: https://github.com/feynmanliang/bachbot.

^5^You can listen to BachBot at: https://soundcloud.com/bachbot.

^6^Performance RNN generates polyphonic compositions of solo piano, and incorporates expressive timing by encoding a flexible rather than strict metrical grid while also allowing the dynamics of each note to vary. Listen to Performance RNN at: https://magenta.tensorflow.org/performance-rnn.

^7^FlowMachines, by Sony Computer Science Laboratories in Paris, is a European Research Council funded project. Unfortunately, it is not an open source project. However, more information on their project, including DeepBach (FlowMachines take on BachBot) can be found at: http://www.flow-machines.com/.

^8^You can listen to “Daddy's Car” at: https://www.youtube.com/watch?v=LSHZ_b05W7o.

^9^Remember, the melody is incorporated as part of the generated leadsheet, as are the instructions for which notes to include in the harmonies, but not necessarily which voice those notes should be assigned to the harmonies—e.g., the guitar or the bass.

^10^Listen to Cybraphon at: https://www.youtube.com/watch?v=wDyabLAzKuo.

^11^Learn more about Shimon and listen to some of its music at: https://www.shimonrobot.com/.

^12^An important consideration, though beyond the scope of this article to discuss in detail, is that designers have seemingly assumed that machine learning is the correct approach to music-making AIs—indeed, all the AIs discussed here rely primarily or exclusively on machine learning. However, for activities that significantly involve complex mutual prediction (this is true of musical collaboration as well as solo musical improvisation and even to some extent non-improvisational solo performance), “learning” in this sense may not be the correct computational approach and *inference*, relying on state estimation and Bayesian filtering, may be a more appropriate paradigm that has been as-yet under-explored (we thank an anonymous reviewer for bringing up this point).
